# Unveiling Smyd-2’s Role in Cytoplasmic Nrf-2 Sequestration and Ferroptosis Induction in Hippocampal Neurons After Cerebral Ischemia/Reperfusion

**DOI:** 10.3390/cells13231969

**Published:** 2024-11-28

**Authors:** Daohang Liu, Yizhun Zhu

**Affiliations:** 1 School of Pharmacy, Shanghai Key Laboratory of Bioactive Small Molecules, Fudan University, Shanghai 201203, China; dhliu@cdutcm.edu.cn; 2 School of Pharmacy, Macau University of Science and Technology, Macau 999078, China

**Keywords:** Smyd-2, Nrf-2, ischemic stroke, ferroptosis, lipid peroxidation, hinge and latch

## Abstract

SET and MYND Domain-Containing 2 (Smyd-2), a specific protein lysine methyltransferase (PKMT), influences both histones and non-histones. Its role in cerebral ischemia/reperfusion (CIR), particularly in ferroptosis—a regulated form of cell death driven by lipid peroxidation—remains poorly understood. This study identifies the expression of Smyd-2 in the brain and investigates its relationship with neuronal programmed cell death (PCD). We specifically investigated how Smyd-2 regulates ferroptosis in CIR through its interaction with the Nuclear Factor Erythroid-2-related Factor-2 (Nrf-2)/Kelch-like ECH-associated protein (Keap-1) pathway. Smyd-2 knockout protects HT-22 cells from Erastin-induced ferroptosis but not TNF-α + Smac-mimetic-induced apoptosis/necroptosis. This neuroprotective effect of Smyd-2 knockout in HT-22 cells after Oxygen–Glucose Deprivation/Reperfusion (OGD/R) was reversed by Erastin. Smyd-2 knockout in HT-22 cells shows neuroprotection primarily via the Nuclear Factor Erythroid-2-related Factor-2 (Nrf-2)/Kelch-like ECH-associated protein (Keap-1) pathway, despite the concurrent upregulation of Smyd-2 and Nrf-2 observed in both the middle cerebral artery occlusion (MCAO) and OGD/R models. Interestingly, vivo experiments demonstrated that Smyd-2 knockout significantly reduced ferroptosis and lipid peroxidation in hippocampal neurons following CIR. Moreover, the Nrf-2 inhibitor ML-385 abolished the neuroprotective effects of Smyd-2 knockout, confirming the pivotal role of Nrf-2 in ferroptosis regulation. Cycloheximide (CHX) fails to reduce Nrf-2 expression in Smyd-2 knockout HT-22 cells. Smyd-2 knockout suppresses Nrf-2 lysine methylation, thereby promoting the Nrf-2/Keap-1 pathway without affecting the PKC-δ/Nrf-2 pathway. Conversely, Smyd-2 overexpression disrupts Nrf-2 nuclear translocation, exacerbating ferroptosis and oxidative stress, highlighting its dual regulatory role. This study underscores Smyd-2’s potential for ischemic stroke treatment by disrupting the Smyd-2/Nrf-2-driven antioxidant capacity, leading to hippocampal neuronal ferroptosis. By clarifying the intricate interplay between ferroptosis and oxidative stress via the Nrf-2/Keap-1 pathway, our findings provide new insights into the molecular mechanisms of CIR and identify Smyd-2 as a promising therapeutic target.

## 1. Introduction

Strokes have become the second leading cause of death and disability worldwide, following only heart disease, with high mortality and morbidity rates [1,2,3,4]. Approximately 80% of all strokes are ischemic, primarily caused by the focal occlusion or stenosis of intracranial/extracranial arteries [5,6]. Minimally invasive endovascular therapy or thrombolytic drugs to a certain extent can restore blood supply in the brain. However, this blood flow simultaneously brings a series of endogenous and exogenous free radicals resulting from reduction–oxidation (redox) imbalance, eventually leading to brain edema and hemorrhagic transformation [7,8,9]. These neuropathological lesions are known as cerebral ischemia/reperfusion (CIR) injuries. Thus, the intervention of reactive oxygen species (ROS) can effectively salvage the function of the ischemic penumbra.

Ferroptosis, a novel type of PCD, occurs in addition to neuronal apoptosis and necrosis after CIR [10,11]. The pathological hallmark of ferroptosis is the peroxidation of phospholipids containing long-chain unsaturated fatty acids on the cytoplasmic and organelle membranes. This process is triggered by the depletion of intracellular glutathione (GSH) and the collapse of the glutathione peroxidase-4 (GPX-4) system in the presence of ferrous ions [12,13,14]. Nrf-2 negatively regulates neuronal ferroptosis by regulating GSH systems, NADPH regeneration systems, dominant iron storage ferritin, and iron transporter ferroportin [15]. As an essential member of the cap-‘n’-collar (CNC) transcription factor family that belongs to leucine zipper proteins (bZIPs) in mammals, Nrf-2 is expressed chiefly in apparatus with high metabolic activity and vital detoxification functions, especially in the brain [16]. Under physiological conditions, the lysine-rich Neh-2 domain of Nrf-2 interacts with the ETGE (high-affinity) and DLG (low-affinity) motifs of Keap-1 to form inactive dimers in the cytoplasm [17,18,19,20]. Keap-1 bundles the ring box protein-1 (RBP-1) and Cullin-3-Rbx-1-E3 ligase complex through its BTB domain to continuously ubiquitinate and degrade Nrf-2, maintaining Nrf-2 activity at a low level [21,22,23].

Interestingly, the constitutive stabilization of Nrf-2 by Keap-1 is interpreted as the “Hinge-and-Latch” recognition model [17,24]. However, during stroke progression, deleterious factors such as oxidative stress and electrophilic agents can modify critical sulfhydryl groups in Keap-1. Simultaneously, phosphorylated Protein Kinase C-δ (PKC-δ) phosphorylates Nrf-2 at serine residue 40 (Ser-40), inducing a conformational change in the Nrf-2-Keap-1 dimer. This alteration rapidly disrupts the ubiquitin-dependent degradation of Nrf-2, allowing it to dissociate from Keap-1 [25,26]. Once released, Nrf-2 translocates into the nucleus, where it preferentially forms heterodimers with small Maf (sMaf) proteins. These heterodimers bind to antioxidant response elements (AREs) through the bZip domain, initiating the transcription of target genes involved in the antioxidative stress response. This mechanism protects neurons from the gradual and cumulative ROS damage that occurs throughout the stroke cycle [27,28,29]. Moreover, Nrf-2 undergoes various post-translational modifications (PTMs), such as phosphorylation and acetylation, which are critical for its functional regulation. Phosphorylation, in particular, plays a pivotal role in Nrf-2’s release from Keap-1 and subsequent nuclear translocation. Notably, the potential interactions among PTMs—such as adjacent phosphorylated serine/threonine residues and methylated lysine/arginine residues—appear to be mutually exclusive. These post-translational modifications in the Neh-2 domain of Nrf-2 significantly influence its nuclear translocation and functional activity, offering promising avenues for the development of novel therapeutic strategies to mitigate CIR-induced neuronal impairment.

In the central nervous system (CNS), epigenetic modification mechanisms were first revealed in the processes of neuronal development and maturation in the frontal lobe, such as the dynamic regulation of DNA methylation in neurogenesis and the regulation of non-histone protein CpH methylation in neuronal maturation [30,31]. SET and MYND Domain-Containing 2 (Smyd-2) is a unique protein lysine methyltransferase (PKMT) from the Myeloid-Nervy-DEAF1 domain protein family. Smyd-2 is primarily engaged in methylating histone H3 lysine K4 (H3K4) and histone H3 lysine K36 (H3K36) and covalently modifies non-histone proteins [32,33]. While the non-histone methylation mechanisms of Smyd-2 have been extensively studied in contexts such as heart development, skeletal muscle formation, cardiovascular diseases, and tumorigenesis, its role in CNS pathology remains largely unexplored. Notably, Smyd-2 has been implicated in regulating the growth and migration of gonadotropin-releasing hormone (GnRH) neurons in the vomeronasal organ (VNO) of mice [34,35]. Our previous research demonstrated that Smyd-2 might contribute to blood–brain barrier (BBB) disruption during CIR injury [36]. This finding aligns with the broader involvement of other Myeloid-Nervy-DEAF1 domain family members in CNS pathology. For instance, Smyd-1 has been reported to regulate skNAC transcription factors, thereby influencing inflammatory activation in the mouse cortical striatum. Similarly, RNA-binding proteins such as TDP-43, FUS, and TLS can downregulate Smyd-3 precursor mRNA, leading to motor neuron death, neuroinflammatory cascades, and PCD.

Non-histone methylation at the same or adjacent lysine residues introduces complexity into the regulation of pathological processes in vulnerable neurons across various CNS diseases. However, current studies remain too preliminary to definitively delineate the precise role of Smyd-2 in the brain. It is unclear whether Smyd-2, like its homologous family members, participates in CIR progression through endogenous PCD regulatory mechanisms. The alterations in non-histone lysine methylation sites and the extent of methylation saturation by Smyd-2 under CIR stimuli also require further validation. Understanding how these dynamic methylation events affect the function of specific non-histone proteins in CIR is a key area for future research. Nuclear-translocated proteins, such as Nrf-2, contain several positively charged lysine residues in their Neh-2 domain that may interact or compete with Smyd-2. Methylation at these lysine residues could potentially influence Nrf-2’s subcellular localization and stability by modulating its phosphorylation-dependent activation or ubiquitin-mediated degradation. Thus, we aim to offer a more detailed investigation of the role and mechanism of Smyd-2 in CIR, highlighting the relationship between Smyd-2 and ferroptosis and unraveling the molecular basis of Nrf-2 ubiquitylation, phosphorylation, and nuclear translocation.

To address the above gaps, our study systematically investigates the role of Smyd-2 in ferroptosis regulation and its interaction with the Nrf-2/Keap-1 signaling pathway during CIR. Using both in vivo (MCAO model) and in vitro (OGD/R model) systems, we explored Smyd-2’s expression patterns and its impact on hippocampal neuron survival. Central to our investigation is the interplay between the Smyd-2-mediated lysine methylation of Nrf-2 and its phosphorylation and nuclear translocation, key processes for anti-lipid peroxidation and redox balance. Through gene targeting approaches and pharmacological interventions, we analyzed the modulation of ferroptosis by Smyd-2 and its influence on neuronal viability, unveiling a novel epigenetic mechanism that exacerbates ferroptosis under CIR conditions. Our findings highlight Smyd-2’s potential as a therapeutic target for ischemic stroke, advancing our understanding of ferroptosis and oxidative stress in neuronal injury.

## 2. Materials and Methods

### 2.1. Chemical Reagents

Radio immunoprecipitation assay (RIPA), phenylmethanesulfonyl fluoride (PMSF), phosphatase inhibitor, loading buffer, tween-20, glycine, BCA protein assay kit, diethylpyrocarbonate (DEPC), sodium dodecyl sulfate (SDS), and goat serum were purchased from Beyotime Biotech, Shanghai, China. Bovine serum albumin (BSA), goat serum, paraformaldehyde (4%), phosphate-buffered saline (PBS), sodium chloride, isopropanol, β-mercaptoethanol (BME), and Anti-Fluorescence Quenching DAPI were purchased from Servicebio Technology Co., Ltd., Wuhan, China. DCFH-DA fluorescent probe and nuclear/cytoplasmic protein extraction kits were purchased from Yeasen Biotech, Shanghai, China. Enhanced chemiluminescence (ECL) and BODIPY 581/591 C11 fluorescent probe were purchased from Millipore, Bedford, MA, USA. Trizol, phenol-chloroform, 2, 3, 5-triphenyl tetrazole chloride (TTC), and Triton-100 were purchased from Sigma-Aldrich, USA. ML-385, cycloheximide (CHX), and DDO-7263 were purchased from MedChemExpress (MCE) Co., Ltd., USA. Fluoro-Jade B^®^ (FJB) fluorescence probe was bought from QiMing Biotechnology Co., Ltd., Shanghai, China. Antibodies (Rabbit) against Smyd-2, FTH-1, COX-2, GPX-4, GAPDH, HO-1, Keap-1, Nrf-2, histone H-3, and methylated lysine were obtained from Proteintech company, Wuhan, China. Antibodies (Rabbit) containing 15-LOX, NQO-1, PGC-1α, PKC-δ, phosphorylated Nrf-2 (p-Nrf-2/Ser-40), phosphorylated PKC δ (p-PKC-δ/Ser359), and the secondary antibodies were provided by Abcam, Cambridge, UK. Antibodies (Mouse) against Smyd-2 were obtained from Santa Cruz Biotechnology, MA, USA. EndoFectin™ Max was provided by iGene Biotechnology Co., Ltd., Guangzhou, China. Annexin V–FITC/PI kits were purchased from Applygen Technologies Inc., Beijing, China. Erastin was obtained from Selleck Chemicals, Houston, TX, USA.

### 2.2. In Vivo Experiments and Grouping

Male C57BL/6J mice aged 6–8 weeks with a weight range of 20 ± 5 g were purchased from Beijing Vital River Laboratory Animal Technology Co., Ltd. The mice were housed in a specific-pathogen-free (SPF) facility at the Animal Experiment Center of the School of Pharmacy, Fudan University. They were kept in individual cages with ad libitum food and water. The temperature and humidity were strictly controlled at 23 ± 2 °C and 50–60%, respectively, with a 12 h light/dark cycle to maintain the circadian rhythm of the mice. The mice were acclimatized in the SPF facility for one week before the start of the experiments. All laboratory animal procedures were conducted in accordance with the Laboratory Animal Operation Procedures and the Laboratory Animal Management Regulations of the Laboratory Animal Center, School of Pharmacy, Fudan University. The mice were fasted and deprived of water for 12 h before surgery. The mice were randomly divided into sham, CIR, and respective medication groups, with 5 to 6 mice in each experimental group.

### 2.3. Establishment of Middle Cerebral Artery Occlusion (MCAO) Model in Mice

The middle cerebral artery occlusion (MCAO) model is widely employed to study the pathophysiology of CIR due to its ability to replicate the key pathological features of ischemic stroke and subsequent reperfusion injury [37]. Wild-type male C57BL/6 mice (aged 8–10 weeks) were fasted for 12 h with free access to water before MCAO. Mice were anesthetized with 0.3% pentobarbital sodium (0.1–0.2 mL/10 g body weight). A surgical incision was made along the midline of the neck to expose the bilateral carotid arteries. The sternocleidomastoid muscle was located and separated from the muscle and fascia, fully exposing the common carotid artery (CCA), external carotid artery (ECA), and internal carotid artery (ICA). The CCA was ligated at the proximal end with a suture, and the ICA was clamped with a vascular clip. The ECA was opened using ophthalmic scissors near its bifurcation with the CCA. A silicone thread (Jialing Biotech, Guangzhou, China) was inserted into the middle cerebral artery (MCA) at a horizontal angle of 45 degrees. The silicone thread was advanced approximately 2 cm into the MCA, effectively blocking blood flow for 1 h. The silicone thread and the surgical suture clamping the CCA were removed to restore blood flow to the ipsilateral hemisphere of the mice. The mice’s body temperature was kept constant during the surgery. Tissue samples were collected according to the specified reperfusion times following the MCAO.

### 2.4. Lateral Ventricle Localization Injection of Inhibitors and Adeno-Associated Virus in Mice

The mice were anesthetized and fixed by clamping their upper incisors onto a crossbar and stabilizing the nose bar by turning a knob while gently placing the ear bars in the mouse’s ear canals and adjusting them to keep the skull level. Adhesions to the meninges were removed with hydrogen peroxide to identify the Bregma point. One of the injection sites was located 2.5 mm to the right of the Bregma point. The neuronal promoters and inhibitors for the expression of the mouse Smyd-2 were packaged into recombinant adeno-associated virus (serotype 9) vectors (Genomeditech, Shanghai, China). The mice were transduced with adeno-associated virus–Smyd-2 mixed with saline at a rate of 2 μL/min, with 2 μL per mouse, 10 days before MCAO modeling. Sham mice received an equal amount of control viral vector for the same duration. ML-385 was injected into the lateral ventricle at a dose of 40 pmol/4 μL 24 h before MCAO modeling. After injection, the syringe needle was left in place at the injection site for 3 min before being slowly removed to promote the absorption of the adeno-associated virus and inhibitors.

### 2.5. Evaluation of Neurological Function

The Adhesive Tape Removal Test was used to assess sensorimotor deficits in mice [38]. The experimental mice were taken out, and a 4 mm diameter medical adhesive tape was randomly attached to both forelimbs. In normal mice, the tape removal process is typically completed within approximately 20 s. The tape removal time was measured at specific reperfusion time points following MCAO. Times exceeding 180 s were considered invalid and excluded from analysis.

The Corner Test assessed unilateral sensory and motor deficits in mice [39]. The experimental mice were placed between two square plates (30 × 30 × 1 cm^3^) connected at a 30° angle with an opening at the other end for the mouse to enter smoothly. The spatial obstruction prompts the mice’s forward and upward reflex action, followed by a turn to face the open end. The probability of turning left or right in normal mice is almost equal, while MCAO/R mice tend to roll over to the contralateral side. The testing was conducted at specific time points according to the reperfusion time point after MCAO. Attempts where the mouse failed to turn upright were not counted toward the experimental trials.

### 2.6. Evaluation of Cerebral Infarct Size

The 2,3,5-Triphenyl Tetrazolium Chloride (TTC) staining method was used to determine the infarct volume in mice following MCAO [40]. The brain tissue was quickly removed and frozen at −80 °C for 5 min after reperfusion. The tissue was then solidified and sliced along the coronal plane on ice, with a slice thickness of 1 mm. The brain tissue slices were incubated in preheated TTC solution at 37 °C to ensure adequate staining. Subsequently, they were fixed in 4% paraformaldehyde solution at 4 °C for 24 h and then photographed. The infarct area (%) was calculated as the infarct volume (white infarct lesion) divided by the total brain volume, multiplied by 100% through Image Pro Plus (IPP) 6.0 software.

### 2.7. Isolation of Nuclear Protein and Cytoplasmic Protein

A mixture of cell lysis buffers A and B from a cell nuclear/cytoplasmic protein extraction kit was prepared in a 20:1 ratio, and PMSF was added to the mixture to a final concentration of 1 mM. The hippocampus was separated and weighed, and a lysis buffer was added to the tissue in a ratio of 200 μL per 60 mg of hippocampal tissue. Magnetic beads were applied to homogenize the mixture with a homogenizer for 30 s at 4 °C, repeated four times. The mixture was then incubated on ice for 15 min and centrifuged at 2000 rpm for 5 min at 4 °C. The precipitate was mixed with cell lysis buffer A containing PMSF at a ratio of 1:20 for 15 min. Cell lysis buffer B was added to the mixture at a ratio of 10 μL per 100 μL of the precipitate. The mixture was then centrifuged at 12,000 rpm for 15 min at 4 °C. The supernatant was collected and subsequently incubated with cell nuclear protein extraction buffer C mixed with PMSF. The mixture was centrifuged at 12,000 rpm for 15 min at 4 °C, and the supernatant was collected in 1.5 mL centrifuge tubes.

### 2.8. Western Blot

Separation and concentration gels were prepared at appropriate concentrations based on the molecular weight of the target protein. Protein loading was conducted according to experimental groups. Gels and electrophoresis apparatus were assembled for electrophoresis at 80 V. The voltage was then increased to 120 V, and electrophoresis was continued for an additional hour with a constant voltage once the clear separation of the protein bands was observed. The proteins were then transferred to a polyvinylidene fluoride (PVDF) membrane (Millipore, USA) based on the target protein’s molecular weight, with a constant current of 250 mA being applied. After completing the protein transfer, the membranes were washed four times with TBST at room temperature for 5 min each and blocked with 5% BSA at room temperature for 2 h. The membranes were incubated with the primary antibody, diluted with BSA, and left on a shaker (Eppendorf, Framingham, MA, USA) in a refrigerator at 4 °C overnight, followed by four washes with TBST. Next, the membranes were incubated with the secondary antibody specific to the primary antibody species for 1 h and washed four times with TBST. ECL detection was used to visualize the protein bands, and ImageJ was used to analyze the grayscale values of the bands.

### 2.9. Immunoprecipitation (IP)

The beads with methylated lysine and Nrf-2 antibodies were bonded and incubated overnight at 4 °C. The antibody-coupled magnetic beads were then placed in the tissue lysate to allow the antibodies to bind to the proteins of interest overnight at 4 °C. A magnetic rack (Thermo, USA) was used to separate the magnetic bead–antigen–antibody complexes from the protein samples. Subsequently, the collected complexes were washed three times with pre-cooled RIPA buffer and were boiled with a 5× loading buffer protein sample for 5 min at 100 °C to purify the targeted proteins. The supernatant was extracted for electrophoresis. Protein expression was analyzed by Western blot.

### 2.10. Assessment of Cerebral Histopathology

Mice were securely fixed on a mouse board and then underwent thoracotomy. The cannula was slowly inserted into the anterior wall near the apex until it reached the left ventricle. The right atrium was then incised, and an appropriate amount of PBS mixed with heparin was infused into the heart. Next, 4% paraformaldehyde was perfused to fix the brain in situ. The fixed brain was then extracted and placed into a 4% paraformaldehyde solution for another 12 h of fixation, followed by dehydration in a gradient glucose solution, paraffin embedding, and sectioning using a Leica1900 slicer (Leica Microsystems Nussloch GmbH, Nussloch, Germany).

Fluoro-jade B (FJB) stain: Brain tissue slices were first deparaffinized. Background fluorescence was blocked, and contrast was optimized before the slides containing brain tissue slices were incubated in a working solution of FJB. After that, the slides of brain tissues were placed in a Coplin jar to incubate in darkness and low light for 10 min. The degeneration of neurons was observed and analyzed using Nikon immunofluorescence microscopy.

LFB (Luxol fast blue) stain: Brain tissue sections were placed in Luxol fast blue solution and left overnight at 56 °C after deparaffinization and the removal of lipids. The morphology of neuronal myelin was observed under a microscope. After staining, the sections were washed in 95% ethanol to remove excess stain and differentiate the tissue, followed by counterstaining with a solution of cresyl violet to visualize the neuronal nuclei. The myelin appeared stained blue, while other structures, such as the nuclei and cytoplasm, were stained pink by the cresyl violet under the microscope. Images were captured and assessed with a Leica microscopic imaging system.

Perls stain: Perls stain was used to visualize iron accumulation in brain tissue samples. Deparaffinized brain tissue sections were incubated in a mixture of potassium ferrocyanide and hydrochloric acid for 10 min. The sections were washed three times with distilled water for 5 min each time. After that, the sections were stained with neutral red for 1 min and finally dehydrated in absolute alcohol and observed under a microscope. In the presence of ferric iron (Fe^3+^), the potassium ferrocyanide solution reacts to form a blue precipitate (Prussian blue). The staining intensity indicates the concentration of iron in the tissue. The counterstaining with hematoxylin helps to distinguish different cell types and tissue structures.

### 2.11. In Vitro Experiments and Grouping

HT-22 cells were cultured in a cell incubator at 37 °C, 5% CO_2_, and 100% humidity. The culture medium consisted of DMEM (Dulbecco’s Modified Eagle’s Medium) supplemented with 10% FBS (fetal bovine serum), 100 U/mL penicillin, and 100 μg/mL streptomycin. The cells were randomly divided into control, OGD/R, and respective medication groups.

### 2.12. Establishment of Oxygen–Glucose Deprivation/Reperfusion Model in HT-22 Cells

After HT-22 cells were seeded onto culture plates, they were washed with PBS three times and then incubated in glucose-free DMEM. The culture dish was then transferred to an anaerobic chamber to simulate hypoxia for 2 h. Following hypoxia, the glucose-free DMEM was replaced with fresh DMEM, and the plates were placed back into the incubator for reperfusion. The duration of reperfusion was adjusted according to specific experimental requirements [41].

### 2.13. RNA Interference

HT-22 cells in good growth status and at an appropriate density were selected and cultivated for transfection. Cell counting was performed to ensure that the number of cells during transfection was sufficient and that the cell confluence was close to 75% before transfection. EndoFectin™-Max transfection reagent was diluted in DMEM and mixed with an appropriate amount of siRNA at room temperature for 30 min to prepare the transfection complex. The complex was added to the DMEM culture medium without antibodies and then transfected into HT-22 cells for 2 h. Subsequently, a serum-containing culture medium was added to occupy about 50% of the original culture medium volume. Transfection was carried out for 24 h and processed according to experimental requirements.

### 2.14. Measurement of Neuronal Viability

In total, 1000–1500 HT-22 cells were seeded per well in a 96-well plate. The CCK8 assay was performed according to the manufacturer’s instructions after OGD/R. Briefly, 20 μL of CCK-8 solution was added to each 96-well plate well and incubated at 37 °C for 2 h. The optical density (OD) was measured at 450 nm using a Thermo Scientific Microplate Reader (Thermo Scientific, Waltham, MA, USA), and the percentage of neuronal viability was calculated by comparing the background-corrected OD of the treatment group to the control group.

### 2.15. Assessment of Lactate Dehydrogenase (LDH) Release

Following the cell seeding procedure described above, HT-22 cells were pretreated with indicated stimulations. The LDH assay was performed according to the manufacturer’s instructions to detect the content of LDH in the supernatant of HT-22 cells. The OD was measured at 490–500 nm using a Thermo Scientific Microplate Reader (Thermo Scientific, Waltham, MA, USA). LDH release rate (%) = (sample OD − low control OD)/(high control OD − low control OD) × 100%.

### 2.16. Enzyme Assay Kits

The contents of Superoxide Dismutase (SOD), Malondialdehyde (MDA), and GSH in HT-22 cells were determined using enzyme assay kits. The supernatant of each HT-22 cell group was collected by centrifugation at 1000× *g* for 15 min after being subjected to modeling or other treatment conditions. The supernatant was then used to measure the OD value at 450 nm to calculate the SOD, MDA, and GSH content in HT-22 cells. The specific kits used were the Total SOD Detection Kit (product ID: S0101S, Beyotime, China), the MDA Detection Kit (product ID: S0131S, Beyotime, China), and the GSH Detection Kit (product ID: G4305-48T, Servicebio, China).

### 2.17. Determination of the Cellular Lipid Peroxidation

HT-22 cells were seeded in a 6-well plate and treated according to the experimental requirements. The level of reactive oxygen species (ROS) in HT-22 cells was measured using the BODIPY-C11 581/591 probe [42]. The BODIPY 581/591 C11 fluorescent probe was dissolved in a serum-free culture medium to make a stock solution with a 10 mmol/L concentration. After adding 1.5 mL of culture medium containing 1 μmol/L BODIPY 581/591 C11 fluorescent probe, HT-22 cells were incubated in a light-avoiding 37 °C incubator for 30 min. The lipid peroxidation intensity was observed under a fluorescence microscope. Red fluorescence indicates that HT-22 cells are in a reduced state, while green fluorescence indicates that cells are in a highly oxidative state due to the ROS-induced oxidation of the unsaturated butadiene in the BODIPY 581/591 C11 fluorescent probe.

### 2.18. Determination of the Mitochondrial Lipid Peroxidation

HT-22 cells were seeded in a 6-well plate and treated according to the experimental requirements. Mitochondrial ROS in HT-22 cells were detected using the DCFH-DA probe [43]. The DCFH-DA probe was initially diluted to a concentration of 10 μmol/L using serum-free culture medium, and 1.5 mL was added to each well. This process was carried out in the absence of light. Fluorescence was observed using Nikon confocal microscopy, and the intensity of green fluorescence was proportional to the expression level of DCF, the oxidation product of DCFH-DA. Higher levels of DCF resulted in stronger green fluorescence, indicating a greater accumulation of mitochondrial ROS.

### 2.19. Identification of Programmed Cell Death in HT-22 Cells

The occurrence of programmed cell death in HT-22 cells was examined using the Annexin V–FITC assay kit. HT-22 cells were resuspended in 200 μL Binding Buffer. In total, 4 μL of 0.5 mg/mL PI and 2 μL of Annexin V–FITC solution were added to the incubator for 15 min. The confocal fluorescence microscope was employed to count the number of Annexin V-positive cells, PI-positive cells, and Annexin V/PI double-stained cells. Annexin V can label cells undergoing early apoptosis and cell death, while PI labels necrotic cells [44].

### 2.20. Mitochondrial Morphology

The HT-22 cells were plated onto a 24-well plate. The supernatant was discarded, and 500 μL of 2.5% glutaraldehyde fixative was added to the wells to fix HT-22 cells at 4 °C for 12 h. The mitochondrial morphology was observed and compared under the Hitachi HT780 transmission electron microscope (Japan).

### 2.21. Statistical Analysis

Statistical analysis was performed using Prism 8.3. The experimental results are presented as mean ± standard deviation (SD). One-way ANOVA was used to compare the differences between multiple groups. *p* < 0.05 was considered statistically significant.

## 3. Results

### 3.1. OGD/R and CIR Induce an Increase in Smyd-2 Expression in Hippocampal Neurons

**Figure 1:**
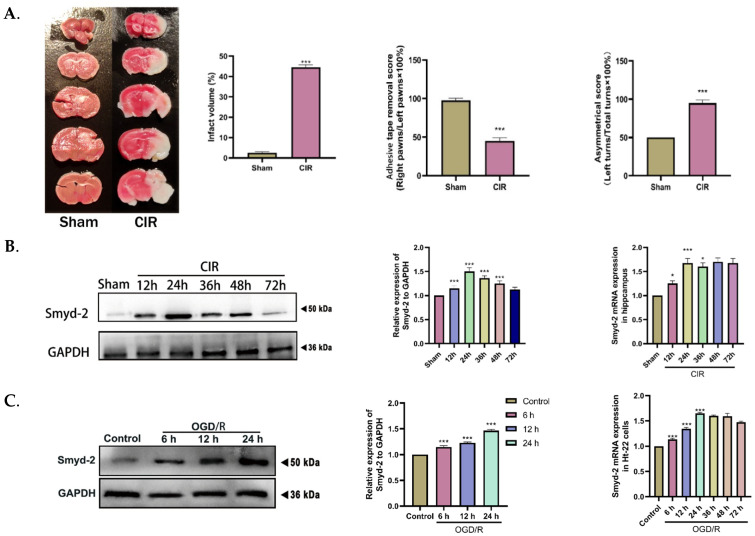
MCAO and OGD/R conduce to Smyd-2 activation in the hippocampus and HT-22 cells. (**A**). Representative images of TTC staining, quantitative analysis of infarct volume, asymmetrical test scores, and the adhesive removal scores in MCAO mice (*n* = 5). (**B**). Smyd-2 expression in mouse brain after CIR (*n* = 5). (**C**). Smyd-2 expression in Ht-22 cells challenged with OGD/R (*n* = 5). * *p* < 0.05 vs. sham group, *** *p* < 0.001 vs. sham group; *** *p* < 0.0001 vs. control group.

We backed up these findings by establishing an OGD/R model for CIR insult. HT-22 cells were subjected to OGD/R as the mice were subjected to CIR. The in vivo experiment results showed that the Smyd-2 protein and mRNA expression reached the highest level and then decreased in a time-dependent manner when mice underwent ischemia for 1 h and reperfusion for 24 h, but there was no significant difference in Smyd-2 mRNA expression after reperfusion for 24 h to 72 h (Figure 1A). Compared with the control group, the Smyd-2 mRNA level and protein expression level of Ht-22 cells gradually increased at the 6th hour after OGD and reached the peak level at the 24th hour (Figure 1B). It is worth noting that the mRNA expression level of Smyd-2 showed no statistical significance from the 24th hour after OGD, which is consistent with the in vivo experiments (Figure 1C). Therefore, we selected Ht-22 cells to undergo OGD for 2 h and then reperfusion for 24 h as the follow-up experimental conditions. The above results show that the protein and mRNA expression of cellular Smyd-2 peaked 24 h after reperfusion.

### 3.2. The Inhibition of Smyd-2 Expression Delays the Progression of CIR Impairment

**Figure 2:**
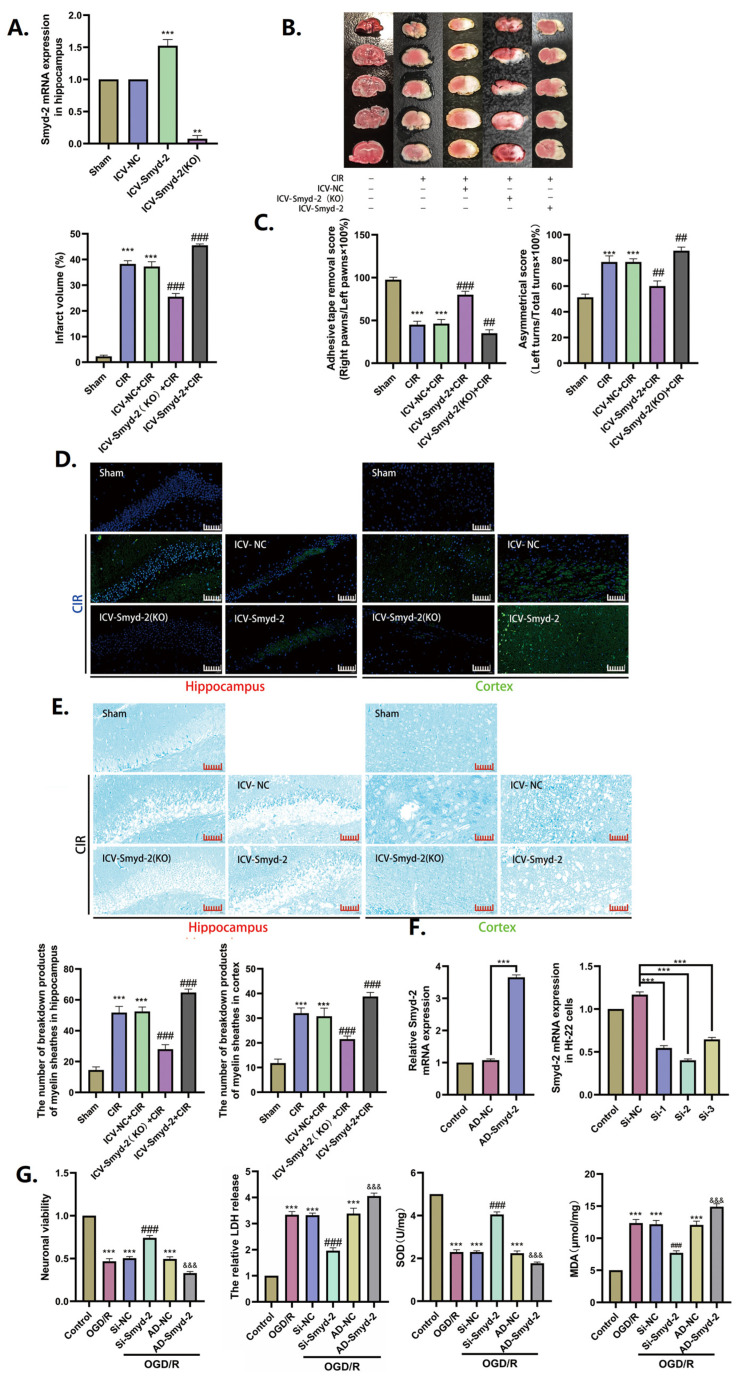
The inhibition of Smyd-2 expression delays the progression of CIR impairment. (**A**–**C**) Effects of Smyd-2 on cerebral infarction volume and neurobehavioral function in MCAO mice (*n* = 5). (**D**) Representative images of FJB staining slices of the hippocampus and cortex; scale bars = 40 μm (*n* = 5). (**E**) Representative images of LFB staining slices of the hippocampus and cortex and quantitative analysis of the breakdown products of myelin sheathes in the hippocampus and cortex; scale bars = 40 μm (*n* = 5). (**F**) The transfection effects of Smyd-2-overexpressing adenovirus and siRNA in Ht-22 cells (*n* = 5). (**G**) The neuronal viability, LDH release, SOD level, and MDA level of Ht-22 cells challenged with Smyd-2 siRNA and Smyd-2-overexpressing adenovirus after OGD/R (*n* = 5). ** *p* < 0.01 vs. sham group, *** *p* < 0.001 vs. sham group; ## *p* < 0.01, ### *p* < 0.001 vs. CIR group and CIR + IVC-NC group; *** *p* < 0.0001 vs. control group; ### *p* < 0.0001 vs. OGD/R group and OGD/R + Si-NC group; &&& *p* < 0.0001 vs. OGD/R group and OGD/R + AD-NC group.

To clarify the mechanism and role of the increased expression of Smyd-2 in the course of CIR, we transfected Smyd-2-overexpressing adeno-associated virus, Smyd-2-silenced adeno-associated virus, and a vector through the intra-cerebroventricular (ICV) injection of mice. The PCR results showed that compared with the sham group, the mRNA expression of Smyd-2 was significantly increased in the ICV-Smyd-2 group, while it was almost not expressed in the ICV-Smyd-2 (KO) group (Figure 2A). These results showed that the ICV injection with adeno-associated virus successfully interfered with the overexpression and silencing of Smyd-2 in the hippocampus of the mouse brain, and this condition and dose was applied to subsequent experiments. The experimental results showed that Smyd-2 knockout could reduce the cerebral infarction volume, improve the neurologic deficit scores, alleviate neuronal demyelination, and reduce the number of degenerative neurons in the hippocampus after CIR (Figure 2B,C,E). However, Smyd-2 overexpression significantly increased the volume of cerebral infarction, significantly decreased the neurologic deficit scores, aggravated neuronal demyelination and degeneration, and increased vacuole-like structures between neurons induced by CIR (Figure 2B,C,E). In vitro, Si-RNA-mediated Smyd-2 knockout (Si-Smyd-2) notably remitted the OGD/R-induced decrease in the viability of HT-22 cells and increased the LDH release (Figure 2G). SOD measures the antioxidant capacity and the neutralization of superoxide radicals, while MDA is typically used to detect lipid peroxidation. The cytosolic SOD levels significantly decreased, while the stable cytosolic metabolite MDA levels markedly increased in OGD/R-induced HT-22 cells (Figure 2G). OGD/R-induced HT-22 cells transfected with adenoviral Smyd-2 expression vectors (AD-Smyd-2) showed double the SOD content and half the MDA content compared to the activity measured in the non-transfection and mock-vehicle groups (Figure 2G). The above data suggests that Smyd-2 is involved in the pathophysiologic procedures of CIR/OGDR. Smyd-2 knockout in hippocampal neurons has neuroprotective potential against CIR/OGDR insult, and Smyd-2 overexpression, in turn, could exacerbate CIR/OGDR impairment.

### 3.3. Smyd-2 Knockout Counteracts the Effect of OGD/R on Lipid Peroxidation in Ht-22 Cells

**Figure 3:**
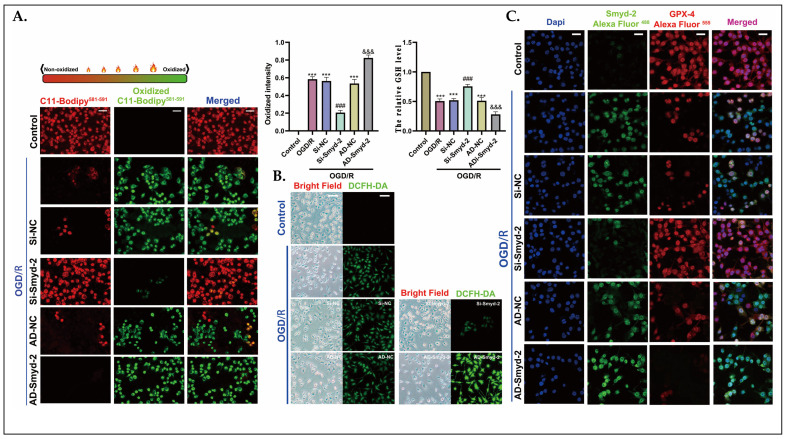
Smyd-2 knockout counteracts the effect of OGD/R on lipid peroxidation in Ht-22 cells. (**A**) BODIPY-581/591-C11 staining was applied to analyze and quantify the effect of Smyd-2 siRNA and adenovirus-mediated Smyd-2 on HT-22 cell ferroptosis challenged with OGD/R. The representative images were obtained with an optical microscope at 400× magnification; scale bars = 50 μm. The GSH level of HT-22 cells challenged with AD-Smyd-2 and Si-Smyd-2 after OGD/R. (**B**) DCFH-DA staining was applied to analyze and quantify the effect of Smyd-2 siRNA and adenovirus-mediated Smyd-2 on HT-22 cell ferroptosis challenged with OGD/R. The representative images were obtained with a confocal microscope at 200× magnification; scale bars = 100 μm (*n* = 6). (**C**) Immunofluorescence method was applied to investigate Smyd-2 and GPX-4 protein expression and localization in HT-22 cells and their relation to neuronal ferroptosis after OGD/R. The representative images were obtained with a confocal microscope at 400× magnification; scale bars = 50 μm. *** *p* < 0.0001 vs. control group; ^###^ *p* < 0.0001 vs. OGD/R group and OGD/R + Si-NC group; &&& *p* < 0.0001 vs. OGD/R group and OGD/R + AD-NC group.

It has been determined that free radicals in HT-22 cells generated by OGD/R oxidized the unsaturated butadiene backbone of the BODIPY-581/591-C11 probe, resulting in a shift in the fluorescence emission from 590 nm to 510 nm, and oxidized DCFH-DA into 2′-7′-dichlorofluorescein (DCF) with a green fluorescent label. Both of these observations suggest that OGD/R can lead to high levels of intracellular ROS and lipid peroxidation products. Si-Smyd-2 in HT-22 cells increased the resistance to lipid peroxidation and reduced the creation of intracellular free radicals when exposed to OGD/R; correspondingly, HT-22 cells transfected with Smyd-2-overexpressing adenovirus appeared to have enhanced lipid peroxidation and generated prodigious quantities of highly reactive free radicals after OGD/R (Figure 3A,B). GSH serves as the detoxifying substrate for GPX-4, which converts cytotoxic phospholipid hydroperoxides into non-toxic alcohols. GPX-4 is a crucial reductase in the pathogenesis of ferroptosis. The results demonstrated that the OGD/R treatment substantially reduced the content of GSH by 50% compared to control HT-22 cells, but OGD/R-induced HT-22 cells transfected with Smyd-2 Si-RNA regained a GSH content of up to 75% (Figure 3A,B). Moreover, double fluorescence staining with Smyd-2 and GPX-4 indicated a significant negative correlation between Smyd-2 expression and GPX-4 activation (Figure 3C). The data above show that the Smyd-2 expression was positively correlated with the ferroptosis index in HT-22 cells after OGD/R.

### 3.4. Smyd-2 Regulates the Abnormality in Neuronal Ferroptosis Caused by CIR

**Figure 4:**
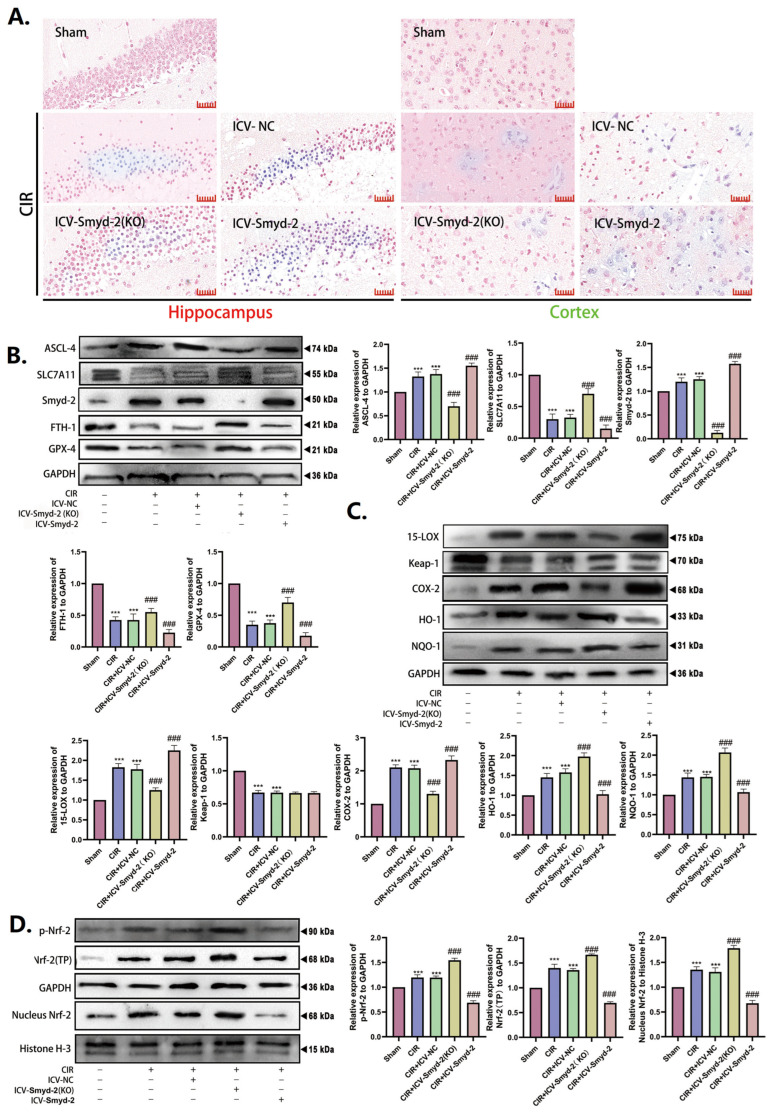
Smyd-2 regulates the abnormality in neuronal ferroptosis caused by CIR. (**A**) Representative images of Perls staining slices of the hippocampus and cortex. Scale bars = 40 μm (*n* = 5). (**B**–**D**) Representative images of Western blot and quantitative analysis of the expression of Smyd-2, GPX-4, FTH-1, SLC7A11, ACSL-4, 15-LOX, COX-2, NQO-1, Keap-1, HO-1, nucleus Nrf-2, *p*-Nrf-2, and Nrf-2 (TP) in the hippocampus. *** *p* < 0.001 vs. sham group; ### *p* < 0.001 vs. CIR group and CIR + ICV-NC group.

Perls blue staining revealed the massive accumulation of ferrocyanide in the CA1 region of the hippocampus in CIR-challenged mice, whereas ICV-Smyd-2 (KO) minimized this accumulation (Figure 4A). Western blot was used to measure ferroptosis-related proteins. The results showed that CIR could increase the expression of Smyd-2, ASCL-4, Nrf-2 (total protein, TP), p-Nrf-2 (phosphorylated Nrf-2), nucleus Nrf-2, 15-LOX, COX-2, HO-1, and NQO-1 and downregulate the expression of SLC7A11, FTH-1, GPX-4, and Keap-1 in mouse hippocampus. In contrast, ICV-Smyd-2 (KO) downregulated the expression of Smyd-2, ASCL-4, 15-LOX, and COX-2 while upregulating the expression of Nrf-2 (total protein, TP), p-Nrf-2 (phosphorylated Nrf-2), nucleus Nrf-2, HO-1, and NQO-1 (Figure 4B–D). Notably, there was no significant difference in the expression of Keap-1 compared to the CIR group.

These results imply that CIR causes ferroptosis in hippocampus neurons, which could be diminished by Smyd-2 knockout, restoring the balance of oxidative stress.

### 3.5. Smyd-2 Knockout Delays the Progression of Erastin-Induced Neuronal Ferroptosis

**Figure 5:**
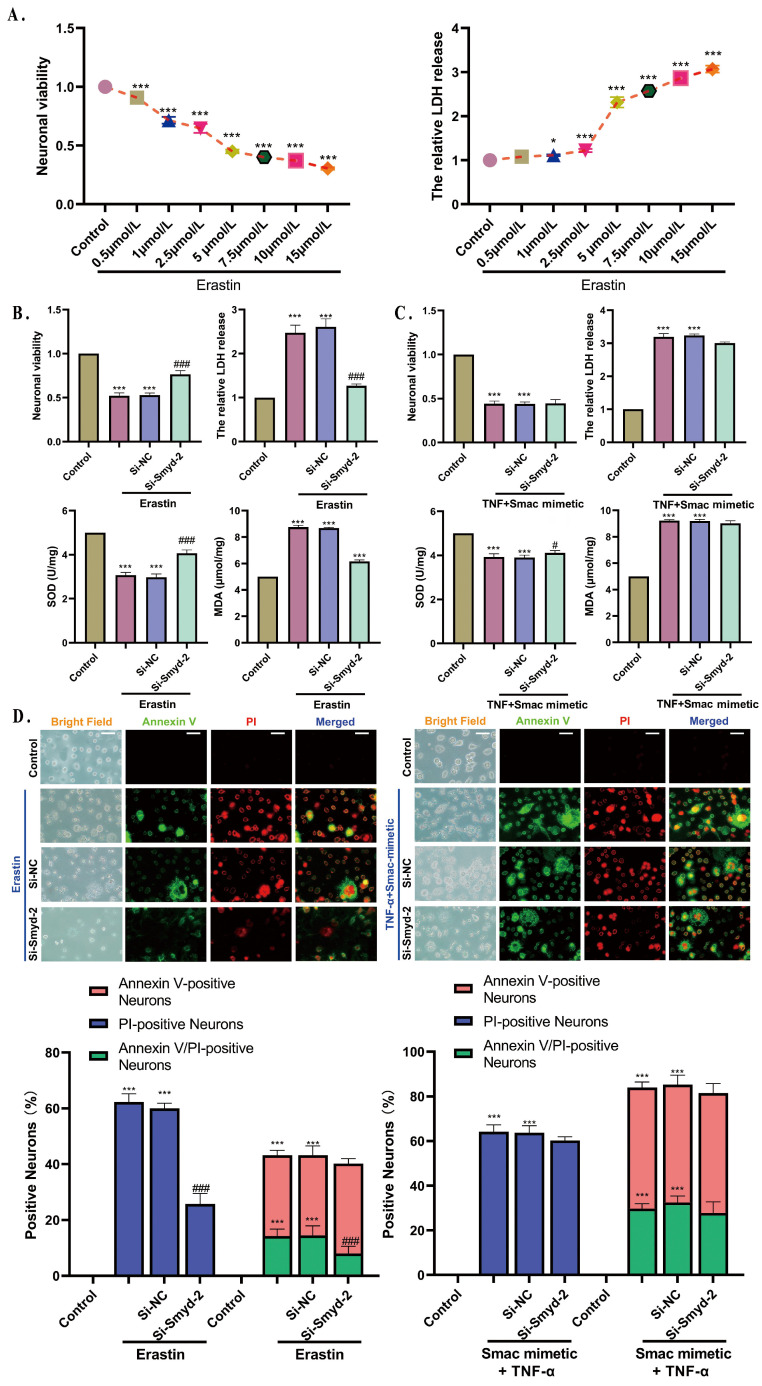
The effect of Smyd-2 overexpression on programmed cell death of HT-22 cells induced by Erastin and Smac mimetic + TNF-α. (**A**) The neuronal viability and LDH release of HT-22 cells challenged with Erastin. (**B**,**C**) The neuronal viability and LDH release of HT-22 cells challenged with Erastin. (c,d,e,f,g,h,i,j) The neuronal viability, LDH release, SOD level, and MDA level of Si-Smyd-2 HT-22 cells challenged with Erastin and Smac mimetic (*n* = 6). (**D**) Annexin V/PI staining was applied to observe and analyze the effect of Erastin and Smac mimetic on different types of programmed cell death in HT-22 cells induced by OGD/R. The representative images were obtained with an optical microscope at 400× magnification; scale bars = 50 μm (*n* = 6). *** *p* < 0.0001 vs. control group, * *p* < 0.05 vs. control group; ### *p* < 0.0001 vs. OGD/R group and OGD/R + Si-NC group, # *p* < 0.05 vs. OGD/R + Si-NC group.

To further illustrate the role of Smyd-2 in neuronal PCDs, Erastin was applied to establish the ferroptosis model by incubating HT-22 cells for 12 h. Additionally, tumor necrosis factor (TNF-α), which can control the activation of the transcription factor NF-κB, was used in conjunction with a Smac mimetic that mimics the function of the pro-apoptotic protein Smac/Diablo. HT-22 cells were incubated with these agents for 15 h to induce apoptosis and necrosis. The optimal concentrations of Erastin, TNF-α, and Smac mimetic to inhibit the growth of HT-22 cells were 5 μMol/L, 100 ng/mL, and 120 nMol/L, respectively, as determined by CCK-8 and LDH colorimetric assays (Figure 5A). Accordingly, the viability and SOD content of HT-22 cells significantly decreased, while the LDH release and MDA content increased in both TNF-α + Smac mimetic and Erastin treatments. Interestingly, knocking down the Smyd-2 expression with siRNA succeeded in driving neuronal viability, increasing SOD content, decreasing MDA content, and minimizing LDH release in the Erastin preconditioning group but failed to have any effect on the neuronal viability, LDH release, and MDA content except for slightly improving the SOD content in the TNF-α and Smac mimetic preconditioning group (Figure 5B,C). Next, propidium iodide (PI) was used to specifically label various types of necrotic cells, as it cannot enter apoptotic cells. Conversely, Annexin V binds to phosphatidylserine (PS), which is translocated from the inner leaflet of the plasma membrane to the cell surface in apoptotic cells, in a Ca^2+^-dependent manner. Based on the established Erastin-induced neuronal ferroptosis and TNF-α + Smac-mimetic-induced neuronal apoptosis/necroptosis, Annexin V/PI immunofluorescence double staining was applied to estimate the PCDs mediated by Smyd-2. Of note, it was validated that both Erastin and TNF-α + Smac mimetic could label HT-22 with Annexin V/PI positivity, with the latter showing a more vigorous intensity of Annexin V-positive fluorescence (Figure 5D). Smyd-2 knockout conspicuously decreased the PI-positive cells without interfering with the Annexin V-positive cells and Annexin V/PI double-stained cells in Erastin-induced HT-22 cells; however, such a decline in PI-positive cells was almost invisible in TNF-α + Smac-mimetic-induced HT-22 cells (Figure 5D). Consistently, Smyd-2 was not significantly involved in excessive apoptosis and necroptosis induced by TNF-α + Smac mimetic but was identified as a critical factor in Erastin-induced neuronal ferroptosis.

### 3.6. The Neuroprotective Effect of Smyd-2 Knockout in OGD/R Can Be Abolished by the Ferroptosis Agonist Erastin

**Figure 6:**
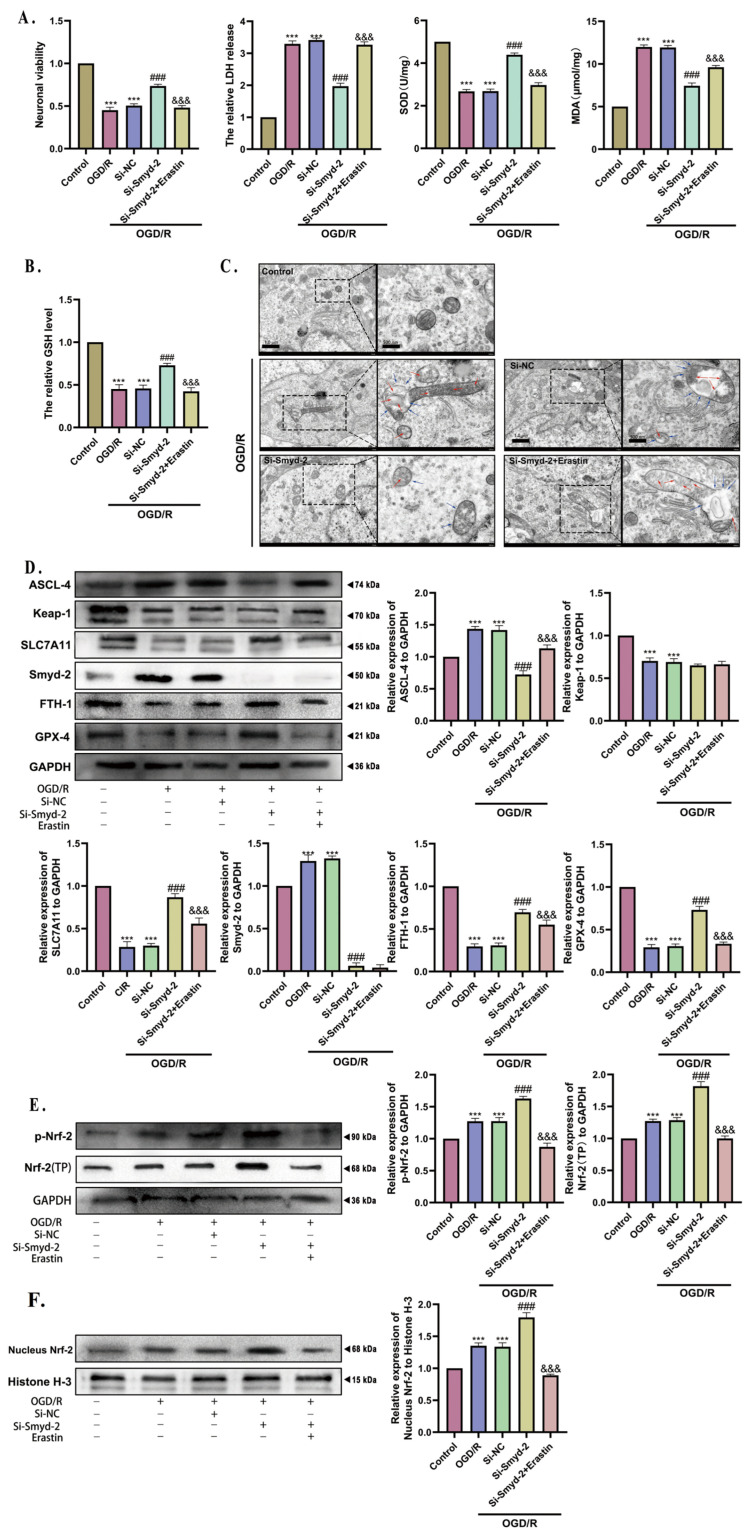
The effect of Si-Smyd-2 combined with Erastin on the ferroptosis of HT-22 cells induced by OGD/R. (**A**) The neuronal viability, LDH release, SOD, and MDA of HT-22 cells challenged with Si-Smyd-2 and Erastin after OGD/R. (**B**) GSH kit was applied to analyze and quantify the effect of the Si-RNA-mediated Smyd-2 and Erastin on HT-22 cell ferroptosis challenged with OGD/R (*n* = 5). (**C**) Pathophysiological and physiological morphologies of mitochondria in each HT-22 cell group were observed by transmission electron microscopy. The red arrows mark the increased electron density of the matrix and fractured and vague cristae. The blue arrows mark vacuoles in mitochondria. The enlarged region bounded by a rectangular dotted box conduces to obtaining a more detailed view of the mitochondria for each experimental condition. The representative images were obtained with an optical microscope at 8k× magnification; scale bars = 1 μm. The representative enlarged images were obtained with an optical microscope at 20k× magnification; scale bars = 500 nm (*n* = 5). (**D**–**F**) Representative Western blots and quantitative evaluation of Smyd-2, SLC7A11, ACSL-4, FTH-1, GPX-4, Nrf-2, Keap-1, p-Nrf-2, and nucleus Nrf-2 expression levels in each HT-22 cell group. Data normalized to the loading control GAPDH are expressed as % of control (*n* = 5). *** *p* < 0.0001 vs. control group; ### *p* < 0.0001 vs. OGD/R group and OGD/R + Si-NC group; &&& *p* < 0.0001 vs. OGD/R + Si-Smyd-2 group.

To further investigate the mechanisms of Smyd-2 in OGD/R-induced hippocampal neuronal ferroptosis, the ferroptosis agonist Erastin (5 μMol/L) was applied to incubate HT-22 cells for 12 h, beginning at the 12th hour after OGD. The experimental results demonstrated that Erastin reversed the antioxidant effects conferred by Si-Smyd-2, as indicated by the recovery of MDA content and LDH release and a reduction in SOD content, GSH, and neuronal viability in HT-22 cells after OGD/R (Figure 6A,B). We then verified the mitochondrial morphology through TEM: the analysis demonstrated that Si-Smyd-2 restored the OGD/R-damaged mitochondrial structure in HT-22 cells, which was primarily manifested as reduced irregular swelling, improved mitochondrial membrane potential, and a more distinct crista structure. However, the maintenance of regular mitochondrial morphology by Si-Smyd-2 was significantly reversed by the application of Erastin (Figure 6C). Finally, Western blot analysis was performed to explore the specific mechanisms by which Erastin counteracts the anti-ferroptosis effects associated with Smyd-2 knockout. The results showed that Erastin impaired the lipid membrane repair system restored by Si-Smyd-2 in HT-22 cells after OGD/R. This impairment was reflected in the downregulation of glutathione reduction system proteins GPX-4 and SLC7A11 and the decreased expression of anti-lipid-peroxidation-related proteins FTH-1, Nrf-2 (total protein), p-Nrf-2, and nucleus Nrf-2, as well as the upregulation of the lipid metabolic enzyme ACSL-4, which converts polyunsaturated fatty acids (PUFAs) into the ferroptosis substrate PUFA–phospholipids (PUFA-PLs) (Figure 6D). Nrf-2 transcriptionally regulates FTH-1 and SLC7A11; it seems that Nrf-2 is the most likely downstream regulatory candidate for Smyd-2. Exceeding our expectations, the expression of Smyd-2 and Keap-1 was unaffected by the Erastin treatment in the Si-Smyd-2 HT-22 cells after OGD/R. These findings indicate that Erastin effectively counteracted the anti-ferroptosis capacity conferred by Smyd-2 knockout in OGD/R-challenged HT-22 cells.

### 3.7. Smyd-2 Knockout Attenuates Neuronal Ferroptosis Induced by CIR/OGDR and Is Mainly Dependent on Nrf-2-Mediated Lipid Metabolic Detoxification

**Figure 7:**
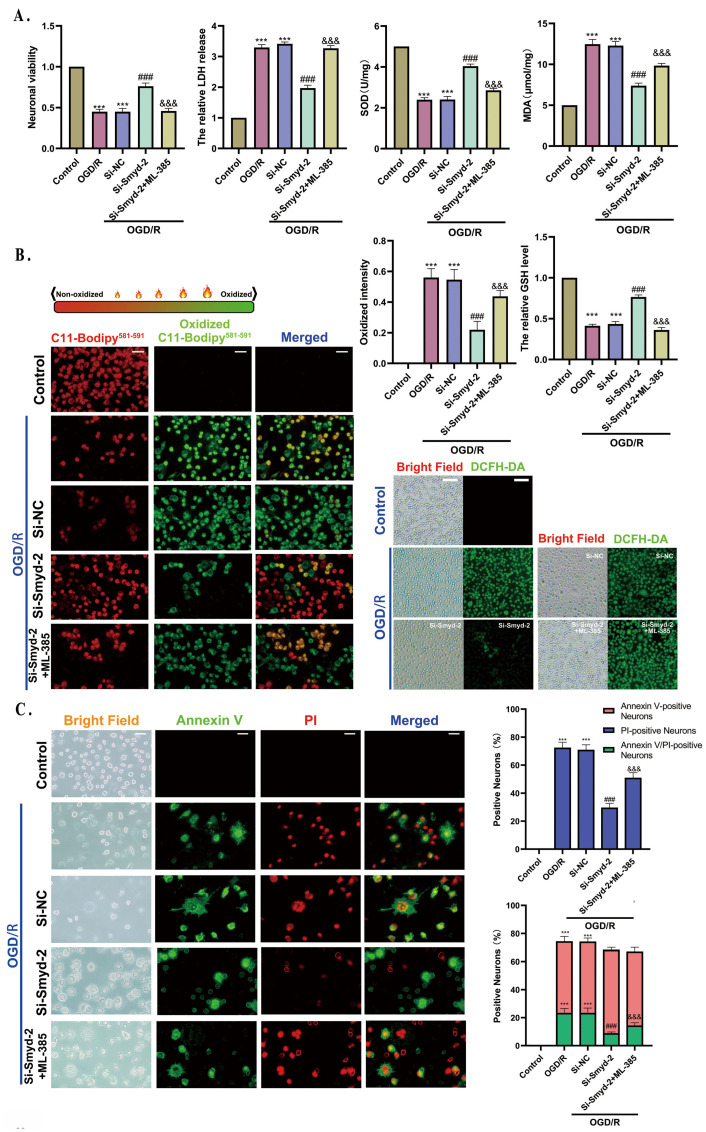

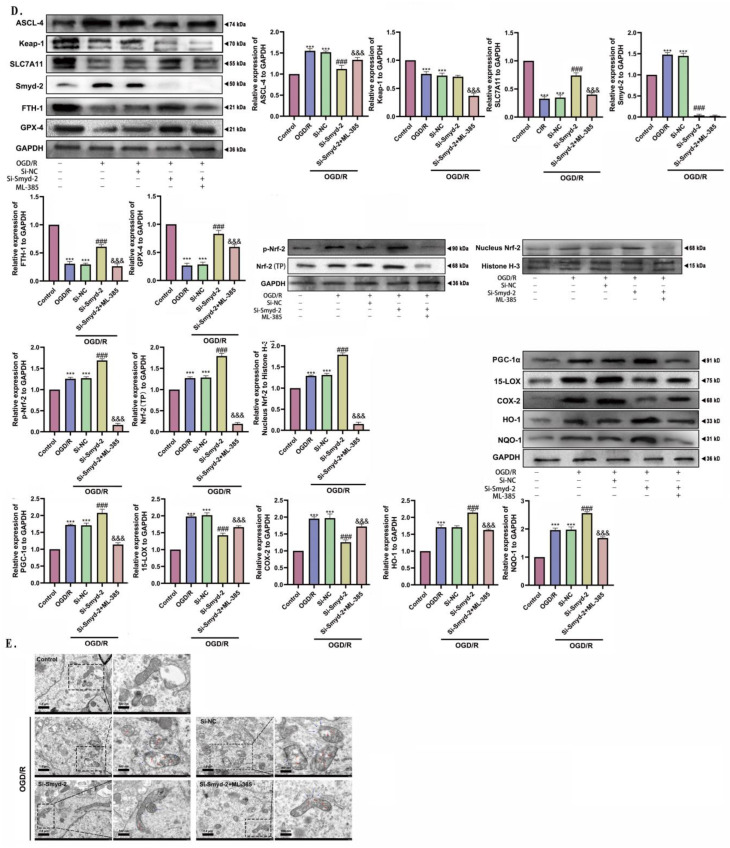
Effects of Smyd-2 (KO) combined with Nrf-2 inhibitor ML-385 on ferroptosis and lipid peroxidation in MCAO mice and OGD/R-induced HT-22 cells. (**A**) The neuronal viability, LDH release, SOD, and MDA of HT-22 cells were challenged with Si-Smyd-2 and ML-385 after OGD/R (*n* = 5). (**B**) BODIPY-581/591-C11 staining, GSH kit, and DCFH-DA staining were used to analyze and quantify the effect of Si-RNA-mediated Smyd-2 and ML-385 on lipid peroxidation of HT-22 cells after OGD/R. The representative images of BODIPY-581/591-C11 staining were obtained with an optical microscope at 400× magnification; scale bars = 50 μm (*n* = 5). The representative images of DCFH-DA staining were obtained with an optical microscope at 200× magnification; scale bars = 100 μm (*n* = 5). (**C**) Annexin V/PI double fluorescence staining was used to study the effect of Si-RNA-mediated Smyd-2 and ML-385 on a different form of programmed cell death in HT-22 cells induced by OGD/R. The representative images were obtained with a confocal microscope at 400× magnification; scale bars = 50 μm (*n* = 5). (**D**) The effect of Si-RNA-mediated Smyd-2 combined with ML-385 on ferroptosis-related proteins in HT-22 cells induced by OGD/R. Representative Western blots and quantitative evaluation of ACSL-4, Keap-1, SLC7A11, Smyd-2, FTH-1, GPX-4, p-Nrf-2, Nrf-2 (TP), nucleus Nrf-2, PGC-1α, COX-2, 15-LOX, NQO-1, and HO-1 expression levels in each HT-22 cell group. Data normalized to the loading control GAPDH are expressed as % of control (*n* = 5). (**E**) Pathophysiological and physiological morphologies of mitochondria in each HT-22 cell group were observed by TEM. The red arrows mark the increased electron density of the matrix and fractured and vague cristae. The blue arrows mark vacuoles in mitochondria. The zoom region bounded by a rectangular dotted box allows a more detailed view of mitochondria for each experimental condition. The representative images were obtained with an optical microscope at 8k× magnification; scale bars = 1 μm (*n* = 5). The representative enlarged images were obtained with an optical microscope at 20k× magnification; scale bars = 500 nm. *** *p* < 0.001 vs. control group; ### *p* < 0.001 vs. OGD/R group and Si-NC + OGD/R group; &&& *p* < 0.001 vs. Si-Smyd-2 + OGD/R group.

We confirmed that Smyd-2 knockout in hippocampal neurons showed robust resistance to CIR/OGDR-induced ferroptosis which was probably engaged in the activation of Nrf-2, but the patterns and mechanisms of how Smyd-2 interacts with Nrf-2 need further validation. ML-385 is a novel and specific inhibitor of Nrf-2, which directly binds to the Neh-1 domain of Nrf-2 to reduce its transcriptional activity. Similar to Erastin’s pro-ferroptosis effects, 2 μmol/L ML-385 scaled up the LDH release and MDA content, leading to reduced viability, GSH content, and SOD levels in Si-Smyd-2 HT-22 cells after OGD/R (Figure 7A). Additionally, ML-385 reduced the amount of ferroptosis in HT-22 cells while ramping up lipid metabolites and ROS accumulation in the mitochondria and cytoplasm of Si-Smyd-2 HT-22 cells after OGD/R (Figure 7B,C,E). It was confirmed that the Nrf-2 inhibitor effectively abolished the antioxidant effect conferred by Smyd-2 knockout. Next, the ML-385 intervention altered the protein expression patterns in Si-Smyd-2 HT-22 cells after OGD/R, leading to a decreased expression of GPX-4, FTH-1, SLC7A11, p-Nrf-2, Nrf-2 (TP), and nucleus Nrf-2, without affecting the expression of Smyd-2. Meanwhile, it is remarkable that the ML-385 treatment decreased the expression of Keap-1, while Erastin did not have much effect on Keap-1 downregulation (Figure 7D).

### 3.8. Smyd-2 Methylates Nrf-2 (Lys-508) to Inhibit OGD/R-Induced Nrf-2 (Ser-40) Phosphorylation and Nuclear Translocation

**Figure 8:**
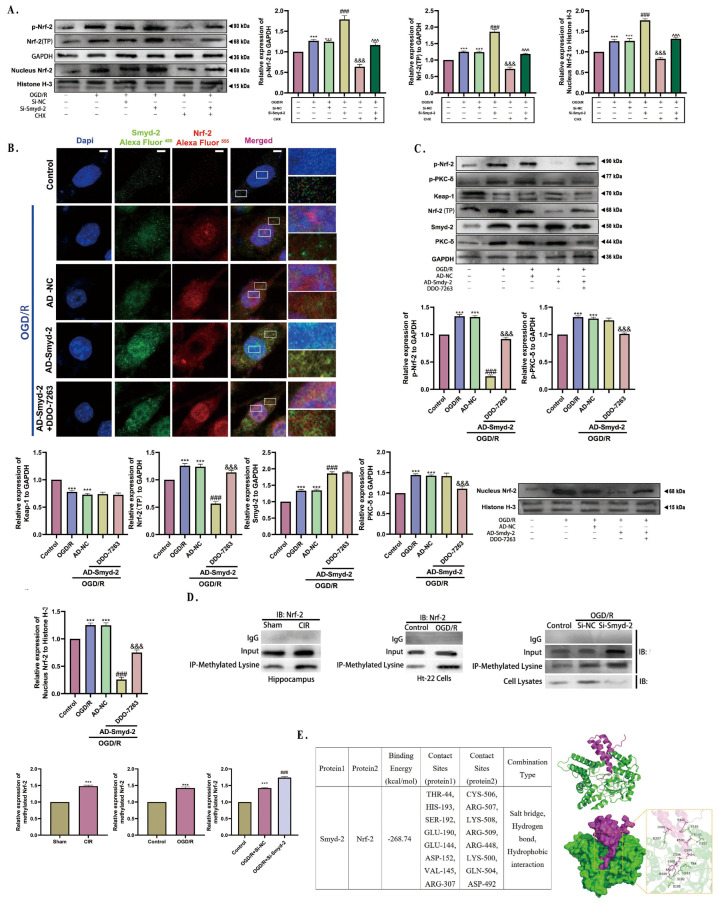
Smyd-2 methylates Nrf-2 (Lys-508) to inhibit OGD/R-induced Nrf-2 (Ser-40) phosphorylation and nuclear translocation. (**A**) Representative Western blot and quantitative evaluation of p-Nrf-2, Nrf-2 (TP), and nucleus Nrf-2 expression levels in each HT-22 cell group. Data normalized to the loading control GAPDH are expressed as % of control. Data normalized to the loading control GAPDH and histone H-3 are expressed as % of control (*n* = 5). (**B**) Confocal images of the localization with immunofluorescence-stained Smyd-2 (green) and immunofluorescence-stained Nrf-2 (red) in various HT-22 cell groups after OGD/R. The zoom region bounded by rectangular boxes represents Smyd-2-Nrf-2 binding in the cytoplasm of HT-22 cells, and the Nrf-2 was transported to the cell nucleus. Scale bars = 10 μm (*n* = 5). (**C**) Representative Western blots and quantitative evaluation of Nrf-2 (TP), p-Nrf-2, nucleus Nrf-2, PKC-δ, p-PKC-δ, Smyd-2, and Keap-1 expression levels in each HT-22 cell group. Data normalized to the loading control histone H-3 are expressed as % of GAPDH (*n* = 5). (**D**) Quantitative analysis of the expression of the methylation level of Nrf-2 (*n* = 5). (**E**) The possible docking sites of two target proteins, Smyd-2/Nrf-2. The binding mode of the complex Nrf-2 with Smyd-2. *** *p* < 0.0001 vs. control group; ### *p* < 0.0001 vs. OGD/R + Si-NC group; &&& *p* < 0.0001 vs. OGD/R group; ^^^ *p* < 0.0001 vs. OGD/R + CHX group. *** *p* < 0.0001 vs. control group; ### *p* < 0.0001 vs. OGD/R group and OGD/R + AD-Smyd-2 group; &&& *p* < 0.0001 vs. OGD/R + AD-Smyd-2 group.

The role of Smyd-2 in regulating the synthesis or degradation of Nrf-2 remains unclear. Firstly, we focused on whether Nrf-2 methylated by Smyd-2 induced Nrf-2 dissociation from the Nrf-2-Keap-1 system or promoted de novo Nrf-2 synthesis. Therefore, HT-22 cells treated with cycloheximide (CHX) (15 μmol/L) were used to inhibit new Nrf-2 synthesis. CHX directly binds to the site on the 80S subunit of the neuronal ribosome to interrupt the translocation of transfer RNA (tRNA), thereby inhibiting the synthesis of most proteins. Notably, Si-RNA-mediated Smyd-2 knockout increased the expression of Nrf-2, p-Nrf-2, and nucleus Nrf-2 after OGD/R, even in the presence of CHX (Figure 8A). This suggests that Smyd-2 has a minimal effect on Nrf-2 synthesis but may influence Nrf-2 degradation pathways.

Subsequently, we inferred that some Smyd-2-associated Nrf-2 degradation mechanisms drive the expression of Nrf-2, p-Nrf-2, and nucleus Nrf-2 induced by OGD/R. It is known that Protein Kinase C-δ (PKC-δ) phosphorylates Nrf-2 at serine residue 40 (Ser-40), promoting its nuclear translocation and preventing Keap-1-Cul-3 ubiquitin E-3 ligase from polyubiquitinating Nrf-2 for degradation. To further investigate this, we used DDO-7263, a potent Nrf-2-ARE activator, which blocks the assembly of the 26S proteasome, inhibiting the degradation of ubiquitinated Nrf-2 and promoting its compensatory upregulation. Laser confocal microscopy was employed to observe the nuclear translocation of Nrf-2 in OGD/R-induced HT-22 cells pretreated with 35 μMol/L DDO-7263. The fluorescence intensity of Smyd-2 and Nrf-2 remained low and localized within the boundaries under normal conditions. The OGD/R challenge boosted the fluorescence intensity of Smyd-2 and Nrf-2, which also manifested Smyd-2 anchored at the neuron cytoplasm and nuclei while activated Nrf-2 mainly functioned in the nuclei (Figure 8B). Smyd-2 overexpression blocked the OGD/R-induced nuclear translocation of Nrf-2, resulting in decreased nuclear expression (Figure 8B). However, DDO-7263 reduced the Nrf-2 nuclear translocation blocking effect of Smyd-2 (Figure 8B). A further investigation was conducted to rule out the possible mechanisms between Smyd-2 and PKC-δ within OGD/R. Western blot analysis showed that the adenovirus-mediated overexpression of Smyd-2 had no effect on the expression of PKC-δ and p-PKC-δ. Moreover, DDO-7263 upregulated the expression of p-Nrf-2, Nrf-2, and nucleus Nrf-2 in Smyd-2-overexpressing HT-22 cells after OGD/R, whereas the expression of Keap-1 and Smyd-2 remained unchanged (Figure 8C). Unexpectedly, DDO-7263 slightly decreased the levels of PKC-δ and p-PKC-δ in the Smyd-2-overexpressing HT-22 cell group induced by OGD/R, suggesting the presence of negative feedback regulation between Nrf-2 and PKC-δ (Figure 8C).

Western blot analysis was performed using an Nrf-2 antibody after immunoprecipitation with anti-methylated lysine in Si-RNA-mediated Smyd-2 knockout HT-22 cells. The results showed that the lysine methylation levels in the Nrf-2 Neh-1 domain increased after CIR/OGD but decreased in Si-RNA-mediated Smyd-2 knockout HT-22 cells after OGD/R (Figure 8D). Meanwhile, HDOCK software (V1.1) was used to set the protein as rigid, the docking contact site as the full surface, and the number of generated conformations to 100. The scoring function was used to select the conformations with the most negative energy, and PyMOL 2.1 software was used for visualization to simulate the Smyd-2-Nrf-2 docking process. The predicted docking site for Smyd-2 on Nrf-2 was the Lys-508 residue in the Neh-1 domain. Importantly, Smyd-2 methylated the Lys-508 residue of Nrf-2 to increase the steric hindrance and create a “cage effect” that inhibits the phosphorylation of Ser-40 on Nrf-2 and also “hijacked” Nrf-2 in the neuronal cytosol with the Keap-1 homodimer facilitating the ubiquitination and proteasomal degradation of Nrf-2, which may be one of the cytoplasmic Nrf-2 stabilizing mechanisms (Figure 8E).

The data above indicate that OGD/R promotes the phosphorylation and nuclear translocation of Nrf-2, while Smyd-2 methylates the Lys-508 residue of Nrf-2 to sequester it in the cytoplasm, thereby exacerbating the ferroptosis regulation during OGD/R.

## 4. Discussion

The pathological and physiological mechanisms underlying ischemic-stroke-induced hippocampal injury have been extensively explored and studied. Lipid peroxidation injury in hippocampal neurons has been identified as a key biomarker for ischemic stroke, accompanied by increased ROS accumulation and reactive nitrogen species, leading to PCD [45,46]. Another risk factor contributing to stroke progression is the disruption of ferrous iron (Fe^2+^) homeostasis in hippocampal neurons. Fe^2+^ can catalyze the removal of hydrogen atoms from the long-chain double bonds of polyunsaturated fatty acids (PUFAs) by lipid peroxides (LPOs) through a Fenton reaction [47,48]. Hippocampal neurons are rich in polyunsaturated fatty acids that are particularly vulnerable to these LPOs, and neutralizing toxic LPOs consumes vast amounts of phase II enzymes, leaving the hippocampal neurons with dire antioxidant factor shortages [49,50,51]. CIR injury wreaks havoc with Fe^2+^ metabolic homeostasis, promotes lipid peroxidation, and ultimately leads to the ferroptosis of hippocampal neurons.

Our previous study showed that the Smyd-2 expression is significantly increased in the cerebral vascular endothelial cells of CIR model mice, suggesting that Smyd-2 may be involved in the destruction of the blood–brain barrier (BBB) caused by CIR. To perfect the role of Smyd-2 in the vast region of the hippocampus that coordinates with the neocortex, we further explored the expression changes and possible mechanisms of Smyd-2 in the hippocampus and cortex by constructing the same mouse model of CIR.

The ischemic phase of cerebral ischemia/reperfusion (CIR) establishes the foundation for neuronal pathology during reperfusion. Upon reperfusion, the restoration of oxygen and glucose supplies generates free radicals, causing significant lipid peroxidation in neuronal membranes [52]. Additionally, CIR induces intracellular calcium overload and releases excitatory glutamate into the synaptic cleft, leading to neurotoxic accumulation in postsynaptic neurons and disruptions in neuronal microcirculation [53,54]. Due to the hippocampal neuron HT-22 cell line’s sensitivity to excitatory glutamate, we selected OGD/R as the preferred in vitro model to simulate CIR [55,56]. Histone methyltransferases, such as mixed-lineage leukemia-2 (MLL2), play critical roles in hippocampal neuroplasticity and neuronal homeostasis. The conditional deletion of MLL2 in mouse hippocampal pyramidal neurons leads to hippocampal-dependent memory loss and severe synaptic plasticity defects, highlighting the close link between these enzymes and cognitive processes [57]. Additionally, Chisholm et al. reported age-related differences in non-histone methylation in an MCAO rat model: histone H3K4 methylation was more abundant than H3K9 in adult astrocytes, while this pattern was reversed in aged rats. Notably, older rats had worse post-MCAO outcomes, potentially due to age-related modifications in astrocyte methylation [58].

In our study, we observed Smyd-2 expression primarily in hippocampal neurons following CIR, with limited expression in the cortex. Notably, Smyd-2 mRNA and protein levels increased in the hippocampus in a time-dependent manner, peaking 24 h after reperfusion, aligning with previous findings that Smyd-2 expression increases in neurovascular endothelial cells in the BBB during OGD/R [36]. However, the expression of Smyd-2 did not remain consistent across models. Unlike in the in vivo results, Smyd-2 mRNA in HT-22 cells stabilized from 24 to 72 h after OGD/R, whereas its hippocampal expression in mice declined after 24 h of reperfusion. This difference may result from the temporal gap between transcription and translation or differences in mRNA degradation timelines in mouse neurons versus HT-22 cells. Our findings suggest that Smyd-2 expression varies across brain regions and experimental conditions in response to CIR. Similarly, its expression patterns differ under various pathological conditions: Smyd-2 is downregulated in neonatal rat myocardial cell injury models induced by cobalt chloride, affecting p53 methylation and stability and promoting myocardial apoptosis [59]. Likewise, in cardiac myocytes (H9c2), Smyd-2 downregulation disrupts myofibril integrity under ROS stimulation [60]. Thus, the upregulation of Smyd-2 in CIR may offer valuable insights into the molecular mechanisms underlying ischemic stroke pathogenesis.

To investigate Smyd-2’s role in CIR, we administered lateral intra-cerebroventricular injections of Smyd-2 adeno-associated virus in mice. In the Smyd-2 knockout group, we observed significant reductions in the cerebral infarction volume and histopathological damage, the absence of hippocampal ferrous ion deposition, and a notable improvement in neurobehavioral scores following CIR. Conversely, in the Smyd-2 overexpression group, the cerebral infarction volume increased markedly, the neurobehavioral scores declined, and there was extensive neuronal damage and diffuse ferrous ion deposition in the hippocampus and cortex. These results suggest that Smyd-2 exacerbates CIR-induced neuronal impairment by promoting ferrous ion accumulation in the hippocampus.

In vitro experiments provided further evidence of Smyd-2’s role. Smyd-2 knockout via adenovirus-delivered siRNA significantly enhanced HT-22 cell viability, reduced mortality, and improved superoxide radical scavenging after OGD/R. In contrast, Smyd-2 overexpression in OGD/R-induced HT-22 cells increased cell mortality, elevated lipid peroxidation, and disrupted redox balance. We speculate that Smyd-2 overexpression compromises anti-lipid peroxidation defenses, leading to the accumulation of toxic lipid peroxides in neurons subjected to OGD/R. This accumulation depletes unsaturated fatty acids, causing brittleness in the neuronal membrane’s lipid bilayer, ultimately reducing membrane fluidity and permeability and resulting in neuronal degeneration and death.

We focus on Smyd-2 knockout increasing the content of GSH, a potent inhibitor of the peroxidation of phospholipids in OGD/R-challenged HT-22 cells. GSH, acting as an auxiliary reductant, facilitates the reduction of strongly oxidizing phospholipid hydroperoxides (PLOOHs) to non-toxic PLOOHs in neurons. This anti-reducing detoxification process is mainly associated with glutathione reductase GPX-4 [61]. GPX-4, an emerging ferroptosis regulator, neutralizes excess intracellular lipid peroxides, preserving lipid bilayer homeostasis [13,62,63,64]. Our immunofluorescence staining showed a negative correlation between Smyd-2 and GPX-4 expression in OGD/R-treated HT-22 cells, indicating that Smyd-2 upregulation may be associated with increased ferroptosis.

In vivo experiments further demonstrated that Smyd-2 (KO) significantly reduces CIR-induced hippocampal ferroptosis by reversing the downregulation of GPX-4, FTH-1, and SLC7A11 while decreasing ACSL-4 expression. FTH-1 has been proven to inhibit ferroptosis through ferritinophagy in the 6-OHDA-induced Parkinson’s Disease (PD) model [65]. SLC7A11 is an essential catalytic subunit of the cystine–glutamate transport system (System XC-). Meanwhile, ischemic stroke produces mass thrombin, simultaneously aggravates calcium overload in the neurovascular unit (NVU), and promotes the phosphorylation of Cytoplasmic Phospholipase A-2 (cPLA-2) to release Arachidonic Acid (AA) [66,67]. ACSL-4 then acetylates AA, incorporating it into phosphatidylethanolamine (PE) and phosphatidylcholine (PC), increasing the susceptibility to lipid peroxidation and thereby driving ferroptosis [68,69,70]. Interestingly, the transcription factor Nrf-2, known for its role in oxidative stress resistance, is upregulated after CIR. The differential expression of Nrf-2 and other anti-ferroptosis proteins in OGD/R-induced HT-22 cells likely reflects variations in the “regulatory time window” of oxidative stress defense mechanisms. Thus, Smyd-2 knockout may alleviate neuronal injury by regulating ferroptosis-related peroxidation reactions and restoring neuronal membrane fluidity.

Another intriguing finding of our research is that Smyd-2 knockout via adenovirus-delivered siRNA in HT-22 cells primarily inhibits Erastin-induced ferroptosis but does not significantly affect tumor necrosis factor-α (TNF-α)/Smac-mimetic-induced apoptosis or necroptosis. Interestingly, although SOD levels correlated well with MDA, we observed a slight increase in SOD content in Smyd-2 knockout HT-22 cells under TNF-α/Smac mimetic induction. This suggests the involvement of other antioxidant stress pathways or a potential temporal lag in lipid oxidation induction relative to ROS removal in this context. A potential alternative explanation for this phenomenon comes from a study by Dhanushka et al., which revealed that ROS can deplete glutathione and oxidize the Cys-13 residue of Smyd-2. This oxidation disrupts Smyd-2’s interactions with Hsp90 and N2A, leading to N2A degradation by MMP-2 and calpain-1, ultimately compromising muscle fiber integrity [60]. These findings imply that Smyd-2 may be negatively influenced by ROS accumulation, suggesting the presence of feedforward and feedback loops involving Smyd-2 in ferroptosis pathways.

Interestingly, Smyd-2 has been shown to promote tumor growth in Ovarian Clear-Cell Carcinoma (OCCC) and Colorectal Cancer (CRC) by inhibiting TNF-induced apoptosis [71,72]. Although these findings differ from our observations, this discrepancy may stem from differences in the activation of pro-death or pro-survival signaling pathways between HT-22 cells and tumor cells under TNF-α/Smac mimetic induction. Moreover, in other ischemic diseases, Smyd-2 inhibits p53-dependent cardiomyocyte death by methylating lysine 382 on p53, thereby reducing its transcriptional activity [59]. Thus, it is evident that in different diseases, non-histone trans-methylases can methylate different sites or poly-methylate the same site, forming complex signaling networks to regulate different types of PCD. Indeed, the prooxidative role of Smyd-2 in other pathological conditions induced neuronal apoptosis model and neuronal necroptosis model remains to be further investigated and established.

Erastin was discovered initially as a small-molecule compound that induces ST and RASV-12 tumor cell death and has since been widely used as a ferroptosis-inducing drug in vitro [73,74]. Unlike other ferroptosis inducers, Erastin mediates ferroptosis in multiple ways, including acting on voltage-dependent anion channels to increase mitochondrial outer membrane permeability, activating ACSL-4 to accelerate the process of lipid peroxidation, reducing cysteine uptake by directly crashing System XC^−^, and upregulating the expression of GSK-3β to decrease Nrf-2 expression in the nucleus [74,75,76]. It has been shown that Erastin-induced ferroptosis in HT-22 cells can be blocked by Zileuton, a LOX-5 inhibitor, via a reduction in cytoplasmic ROS accumulation [77]. We demonstrated that the anti-lipid peroxidation ability and mitochondrial protection conferred by Smyd-2 knockout in OGD/R-damaged HT-22 cells could be reversed by Erastin. Thus, Smyd-2 is a crucial regulator with a critical adjusting function in the processes of neuronal ferroptosis.

Triggering neuronal ferroptosis requires both “conditions” and “opportunities”. The “conditions” are the formation of PUFA-PL and an increment in the labile iron pool (LIP), both of which provide prooxidant iron components for the endogenous Fenton reaction and induce lipid metabolism disorders in the mitochondria [78,79]. The “opportunities” refer to the incapacity of intracellular anti-lipid peroxidation systems, including the previously mentioned cystine–glutamate (System XC^−^), Nrf-2/Keap-1, FSP-1–CoQH-2, and DHODH–CoQH-2 systems. Nrf-2/Keap-1 mainly acts in the cytoplasm and mitochondria [80,81,82]. Our experiments show that Smyd-2 knockout in OGD/R-induced HT-22 cells displays an extraordinary upregulation of Nrf-2, phosphorylated Nrf-2 (p-Nrf-2), and nucleus Nrf-2, also accompanied by deformed or absent mitochondria and neuronal degeneration. The results above indicate that there is likely a link between Smyd-2 and Nrf-2/Keap-1. ML-385 partially reversed the neuroprotective effect of Smyd-2 knockout in the CIR-created hippocampus lesions, characterized by the significantly increased volume of cerebral infarction, more obvious motor impairment, more outstanding cognitive dysfunction, and more heavy ferrous iron accumulation in the hippocampus. Furthermore, we found that the methylation level of lysine residues on Nrf-2 significantly increased after CIR. The regulatory mechanism of Smyd-2 in CIR involves the Nrf-2-Keap-1-ARE pathway. The administration of the Nrf-2 inhibitor ML-385 decreases the expression of anti-ferroptosis proteins in Smyd-2 knockout hippocampus after CIR, including Nrf-2, p-Nrf-2, nuclear Nrf-2, GPX-4, FTH-1, SLC7A11, and NQO-1, while increasing the expression of ferroptosis-related oxidative stress factors 15-LOX and COX-2.

The Nrf-2-Keap-1 pathway plays a central role in maintaining neuronal redox homeostasis and regulating the antioxidative stress response during CIR [83,84,85,86,87]. Under normal conditions, Nrf-2 levels are kept low by forming a complex with Keap-1-Cul-3-E3 ubiquitin ligase, facilitating continuous ubiquitin-mediated degradation. Keap-1 also functions as a cysteine-based sensor for electrophilic reagents and serves as a target for drugs that promote Nrf-2 dissociation. OGD/R stimulation inhibits the proteasomal degradation of Nrf-2, thereby increasing its abundance to counteract LPO [22,23,88]. Notably, Nrf-2 regulates a substantial number of genes involved in oxidative stress defense, with 244 of its 645 basal target genes and 654 inducible direct target genes counteracting LPO [89]. Additionally, Nrf-2 drives the antioxidant responsive element (ARE)-mediated expression of phase II detoxifying enzymes, including HO-1, COX-2, NQO-1, and 15-LOX [90,91,92,93].

Our data indicate that the hippocampal antioxidative stress response is activated after CIR. Smyd-2 knockout in hippocampal neurons further enhances this response, significantly improving both antioxidative capacity and neuronal morphology. Specifically, Smyd-2 knockout favors the Nrf-2-Keap-1-ARE pathway while inhibiting the Nrf-2-Keap-1–ubiquitin pathway, which promotes Nrf-2 degradation via the E3 ubiquitin ligase. By disrupting the “Hinge-and-Latch” model of Nrf-2-Keap-1, Smyd-2 knockout allows more Nrf-2 to escape ubiquitin degradation. Interestingly, Smyd-2 knockout does not affect Keap-1 expression after CIR. This may be attributed to the slower reaction of Keap-1 to electrophiles in the short term. Studies suggest that Keap-1 downregulates its own expression via autophagic degradation mechanisms during prolonged oxidative stress, allowing for sustained Nrf-2 activation [21,94,95]. Despite its critical role in oxidative stress, Keap-1 has limited therapeutic significance in acute disease models such as ischemic stroke, largely due to its bio-refractory properties and long half-life (e.g., 12.7 h in HepG-2 cells) [96,97]. However, these characteristics make Keap-1 a more suitable target for chronic diseases like cancer.

The PTMs of Nrf-2 through phosphorylation or acetylation are adaptable in CIR treatment, which is the essential prerequisite for the nuclear translocation and function of Nrf-2 [98,99]. Keratinocyte growth factor (KGF) phosphorylates Nrf-2 at Ser-40 to promote its activation in endometrial cells [100]. Other cytoplasmic modifiers, including Protein Kinase C (PKC), Mitogen-Activated Protein Kinase (MAPK), Glycogen Synthase Kinase 3β (GSK-3β), and Phosphatidylinositol 3-Kinase (PI3K), also phosphorylate Nrf-2 at serine or threonine residues [101,102,103]. Even within the nucleus, GSK-3β regulates excessive Nrf-2 levels by phosphorylating it, allowing recognition by β-TrCP, which forms an SCFβ-TRCP E3 ubiquitin ligase complex for proteasomal degradation [104]. This regulation shares similarities with Keap-1-mediated degradation; however, the threshold and coordination mechanisms require further investigation. Therefore, exploring PTMs in different Nrf-2 domains offers great potential for understanding and targeting its regulatory mechanisms.

Our study showed that the adenovirus-mediated overexpression of Smyd-2 does not affect the nuclear translocation of Nrf-2 by downregulating the expression of PKC-δ and p-PKC-δ. Instead, Smyd-2 methylates lysine residues in the Neh-2 domain of Nrf-2, increasing the steric hindrance for phosphorylation at Ser-40, which blocks Nrf-2 phosphorylation signal transduction and inhibits its nuclear translocation. The methylation of Nrf-2 decreases the expression of total Nrf-2 protein, phosphorylated Nrf-2, and nucleus Nrf-2; that is, Smyd-2 stabilizes the forward transduction of the Nrf-2-Keap-1-CUL-3–ubiquitin signaling pathway, continuously promoting the ubiquitin-mediated degradation of Nrf-2 to keep the DGR domain of Keap-1 in an unsaturated state that can capture more newly translated Nrf-2 in the cytosols. Smyd-2 “hijacks” Nrf-2 by methylating it in the cytoplasm, leading to its enzymatic hydrolysis and the suppression of Nrf-2 nuclear translocation.

PKC phosphorylates Nrf-2, participating in nuclear transcriptional regulation, which upregulates the expression of HO-1 and NQO-1 while reducing the expression of 15-LOX and COX-2 [105,106]. Previous studies have demonstrated that the dissociation of Nrf-2 is due to the modification of the active cysteine residue of the electrophilic Keap-1 to reduce the activity of the KEAP-1-Cul-3-E3 ubiquitin ligase complex constantly. In contrast, the direct modification of Nrf-2 in the Hinge-and-Latch system is limited to PKC-associated phosphorylation pathways [107,108]. Unexpectedly, Smyd-2 methylates lysine residues between the DLG and ETGE motifs at the Neh-2 domain of Nrf-2 to block its phosphorylation activation. It also stabilizes Nrf-2 in the neuronal cytoplasm and promotes the targeted binding of the Keap1-Cul3-E3 ubiquitin ligase to lysine residues of Nrf-2. Ubiquitinated Nrf-2 is then delivered to the 26S proteasome for degradation. Significantly, AREs mediate the transcriptional induction of the Rbx-1, Cul-3, and Keap-1 genes [109]. This negative feedback regulation prevents the hyperactivation of the Nrf-2-ARE pathway to some extent, but it delays the recovery of the antioxidative stress function in hippocampal neurons following CIR-induced injury.

## 5. Conclusions

This paper provides a detailed description of how Smyd-2 methylates the LYS-508 residue of Nrf-2 to regulate its phosphorylation level, effectively “hijacking” Nrf-2 into the Nrf-2-Keap-1–ubiquitin degradation system. This process induces ferroptosis and lipid peroxidation in hippocampal neuronal membranes and mitochondria as a result of CIR. After ruling out the potential effects of Smyd-2 on TNF-α and Smac-mimetic-induced neuronal apoptosis and necroptosis, we further establish the crucial regulatory role of Smyd-2 in neuronal ferroptosis. Given the constitutive expression of Smyd-2 in hippocampal neurons and its quick-acting Nrf-2-dependent curative pathway, the regimen of Smyd-2-related compounds will be viable clinically against ischemic stroke.

## Data Availability

All data needed to evaluate the conclusions in this paper are present in this paper and available on request.
